# Immunoadsorption to remove ß2 adrenergic receptor antibodies in Chronic Fatigue Syndrome CFS/ME

**DOI:** 10.1371/journal.pone.0193672

**Published:** 2018-03-15

**Authors:** Carmen Scheibenbogen, Madlen Loebel, Helma Freitag, Anne Krueger, Sandra Bauer, Michaela Antelmann, Wolfram Doehner, Nadja Scherbakov, Harald Heidecke, Petra Reinke, Hans-Dieter Volk, Patricia Grabowski

**Affiliations:** 1 Institute for Medical Immunology, Charité—Universitätsmedizin Berlin, Berlin, Germany; 2 Berlin-Brandenburg Center for Regenerative Therapies (BCRT), Berlin, Germany; 3 Department of Nephrology, Charité—Universitätsmedizin Berlin, Berlin, Germany; 4 Center for Stroke Research Berlin, Charité—Universitätsmedizin Berlin, Berlin, Germany; 5 CellTrend GmbH, Luckenwalde, Germany; Cleveland Clinic, UNITED STATES

## Abstract

**Introduction:**

Infection-triggered disease onset, chronic immune activation and autonomic dysregulation in Chronic Fatigue Syndrome/Myalgic Encephalomyelitis (CFS/ME) point to an autoimmune disease directed against neurotransmitter receptors. We had observed elevated autoantibodies against ß2 adrenergic receptors, and muscarinic 3 and 4 acetylcholine receptors in a subset of patients. Immunoadsorption (IA) was shown to be effective in removing autoantibodies and improve outcome in various autoimmune diseases.

**Methods:**

10 patients with post-infectious CFS/ME and elevated ß2 autoantibodies were treated with IA with an IgG-binding column for 5 days. We assessed severity of symptoms as outcome parameter by disease specific scores. Antibodies were determined by ELISA and B cell phenotype by flow cytometry.

**Results:**

IgG levels dropped to median 0.73 g/l (normal 7–16 g/l) after the 4^th^ cycle of IA, while IgA and IgM levels remained unchanged. Similarly, elevated ß2 IgG antibodies rapidly decreased during IA in 9 of 10 patients. Also 6 months later ß2 autoantibodies were significantly lower compared to pretreatment. Frequency of memory B cells significantly decreased and frequency of plasma cells increased after the 4^th^ IA cycle. A rapid improvement of symptoms was reported by 7 patients during the IA. 3 of these patients had long lasting moderate to marked improvement for 6–12+ months, 2 patients had short improvement only and 2 patients improved for several months following initial worsening.

**Conclusions:**

IA can remove autoantibodies against ß2 adrenergic receptor and lead to clinical improvement. B cell phenotyping provides evidence for an effect of IA on memory B cell development. Data from our pilot trial warrants further studies in CFS/ME.

## Introduction

With an estimated prevalence of 0.3%, CFS/ME is a frequent and chronic disease, which is in many cases triggered by an infection. As a result, patients develop a chronic disease characterized by severe fatigue, cognitive dysfunction, flu-like symptoms and massively impaired energy metabolism associated with exertion intolerance [1]. First clear evidence for a pathogenic role of autoantibodies comes from two clinical trials with the monoclonal anti-CD20 antibody rituximab [2, 3]. Upon depletion of CD20+ B cells with rituximab approximately 60% of patients experienced a partial or complete, and in some patients sustained, clinical remission. The delayed onset of response with a median of approximately 4 months in both trials suggests that clinical effects are not mediated by direct depletion of CD20+ B cells, but rather of short-lived antibody-producing plasma cells arising from CD20+ memory B cells, followed by subsequent wash-out of autoantibodies. We have demonstrated recently elevated antibodies against ß2 adrenergic receptors (ß2) and muscarinic M3 and M4 acetylcholine receptors (M3/M4) in a subset of CFS/ME patients in accordance with previous studies [4–6]. Antibodies to ß2 and M3 receptors had been reported in various other diseases including dilatative cardiomyopathy, postural tachycardia, regional pain syndrome, Alzheimer, Sjögren’s syndrome, asthma and others [7]. In patients receiving rituximab we observed a sustained decline of pretreatment elevated ß2 antibody levels in clinical responders to rituximab treatment [4].

Immunoadsorption is an apheresis procedure to remove specific proteins from a patient’s plasma [8]. The plasma is passed through an absorber which selectively binds IgG and can be regenerated and reloaded during processing of the plasma allowing a highly effective removal of IgG with little side-effects. IA was shown to result in moderate to marked clinical improvement in various types of autoimmune disease including dilatative cardiomyopathy and neurological diseases associated with autoantibodies [9–15]. ß1 autoantibodies are frequently found in dilatative cardiomyopathy, and follow up data from IA studies show long-term decrease of these antibodies associated with an improved clinical outcome [9, 10].

Here we describe a first prospective observational IA study in 10 patients with infection-triggered CFS with elevated ß2 antibodies.

## Materials and methods

### Patients

Patients were diagnosed at the outpatient clinic for immunodeficiencies at the Institute of Medical Immunology at the Charité Berlin between 2014 and 2016. Diagnosis of CFS/ME was based on Canadian Criteria [1] and exclusion of other medical or neurological diseases which may cause fatigue. Further infection-triggered disease onset, disease severity according to the Bell scale of ≤ 50 of 100 [16], and elevated levels of ß2 antibodies were required for study inclusion.

### Study protocol

The IA was performed using Globaffin columns containing peptides specifically binding IgG (Fresenius). IA was conducted in 5 cycles on days 1–3 and 6–7 with 2 to 2.5-fold plasma volume filtered. After the 5^th^ IA cycle all patients received 25 g IgG i.v. (Octagam, Octapharma). Immunoadsorptiion is licensed in Germany for the treatment of patients with autoantibodies. We performed an observational study to document course of antibodies and symptoms during and after therapy. The study was approved by the Ethics Committee of Charité Universitätsmedizin Berlin in accordance with the 1964 Declaration of Helsinki and its later amendments. All patients gave written informed consent.

### Assessment of antibodies and IgG

Antibodies against ß2 and M3/M4 receptors were determined by CellTrend GmbH, Luckenwalde, Germany using ELISA technology. Pre and post treatment samples were analyzed in the same assay run. Elevated antibodies were defined as being larger than the 90% percentile of a healthy control group [4]. Total serum IgG and IgG against tetanus and pneumococcal polysaccharide (PcP) were determined at the Charité diagnostics laboratory Labor Berlin GmbH.

### B cell phenotyping

Flow cytometry analyses were performed from whole blood samples after lysis of erythrocytes with BD FACS Lysing Solution (BD Bioscience, San Jose, USA). The antibody panel used contained: anti-CD3-Pacific Blue (PB), anti-CD19-phycoerythrin (PE)/Cy7, anti-CD27-fluorescein isothiocyanate (FITC), anti-CD21 PE, anti-CD38-Alexa Fluor700, anti-CD24-PerCP/Cy5.5, anti-IgM-allophycocyanin (APC) and anti-IgD-APC/Cy7 (BioLegend, USA). The LIVE/DEAD Fixable Aqua Dead Cell Stain Kit (for 405 nm excitation, Invitrogen, life technologies, Carlsbad, USA) was used for life/dead discrimination of cells. Following a washing step antibody mix was applied to 100 μl of blood sample and incubated for 30 minutes at 4°C. After washing the cells with PBS supplemented with 2% Flebo-γ, samples were measured on the cytometer LRS II (BD, Franklin Lakes, USA).

### Assessment of symptoms by scores

The presence and severity of symptoms was assessed before IA using a questionnaire developed by Fluge *et al*. [3]. Symptoms were classified according to a scale (1–10; 1: no symptoms; 5: moderate symptoms; 10: very severe symptoms). At follow-up, the patients recorded monthly the change in symptoms compared to baseline (0: major worsening; 1: moderate worsening; 2: slight worsening; 3: no change from baseline; 4: slight improvement; 5: moderate improvement; 6: major improvement). The fatigue score was calculated as the mean of fatigue, malaise after exertion, need for rest and daily functioning, cognitive score as mean of memory disturbance, concentration ability and mental tiredness and immune score as mean of painful lymph nodes, sore throat and flu-like symptoms.

Further at baseline, and then monthly, fatigue and cognitive impairment were assessed by FACT-F a 13 item questionnaire assessing fatigue with 52 (no fatigue) to 0 (severest fatigue) [17].

### Assessment of muscle strength and endothelial function

Isometric pinch strength of the stronger hand was analyzed using the pinch dynamometer (Saehan Corporation, Korea). The highest of three pinch measurements was used for analyses [18].

Peripheral endothelial function was evaluated by pulse arterial tonometry (PAT) device (EndoPAT-2000, Itamar, Israel). Assessments were performed under standardized conditions after at least 15 minutes of supine rest in a quiet, air-conditioned room. Endothelial dysfunction (ED) was defined by reactive hyperemia index (RHI) ≤1.8 as described previously [19].

### Statistical analysis

Statistical data analyses were done using the software GraphPad Prism 6.0. Nonparametric statistical methods were used. Continuous variables were expressed as median and interquartile range (IQR). Univariate comparisons of two independent groups were done using the Mann-Whitney-U test or Fisher’s exact test, comparisons of two dependent groups were done using the Wilcoxon matched-pairs signed-rank test. A two-tailed p-value of <0.05 was considered statistically significant.

## Results

### Patient characteristics and IA treatment

All patients had chronic severe infection-triggered CFS/ME. Disease severity was assessed by Bell scale and patient characteristics are shown in Table 1. Prior to IA all patients had elevated antibodies against β2, in addition 7 patients against ß1 adrenergic receptors and 6 patients against both M3 and M4 acetylcholine receptors.

**Table 1:** Patients Characteristics.

Pat	sex/age	CFS/ME onset	Disease severity Bell score	Elevated autoantibodies before IA
1	f/42	2011	50	ß2
2	f/59	2011	30	ß1/2, M3/M4
3	f/34	2005	40	ß1/2, M3/M4
4	m/48	2000	20	ß1/2, M3/M4
5	f/37	2012	40	ß1/2, M3/M4
6	f/31	2002	50	ß1/2, M3/M4
7	f/38	1998	20	ß1/2, M3/M4
8	f/30	2005	10	ß2
9	m/46	2013	40	ß2*
10	f/42	2008	30	ß1/2

*ß2 elevated 3 months before IA but not at the day before IA.

Five cycles of IA were conducted on days 1–3 and 6–7. In all patients, there was a drop of fibrinogen, and in 9 out of 10 also a decrease of albumin levels below the normal range. There was one mild bleeding episode in one patient. In one patient albumin was substituted due to dizziness. Immediately after the 5^th^ IA all patients received 25 g IgG (i.v.). Patients 7 and 10 reported worsening of symptoms due to the exertion of the daily IA and the hospital stay. Both patients therefore had only 4 IA cycles.

### Course of IgG and autoantibodies

**Fig 1:**
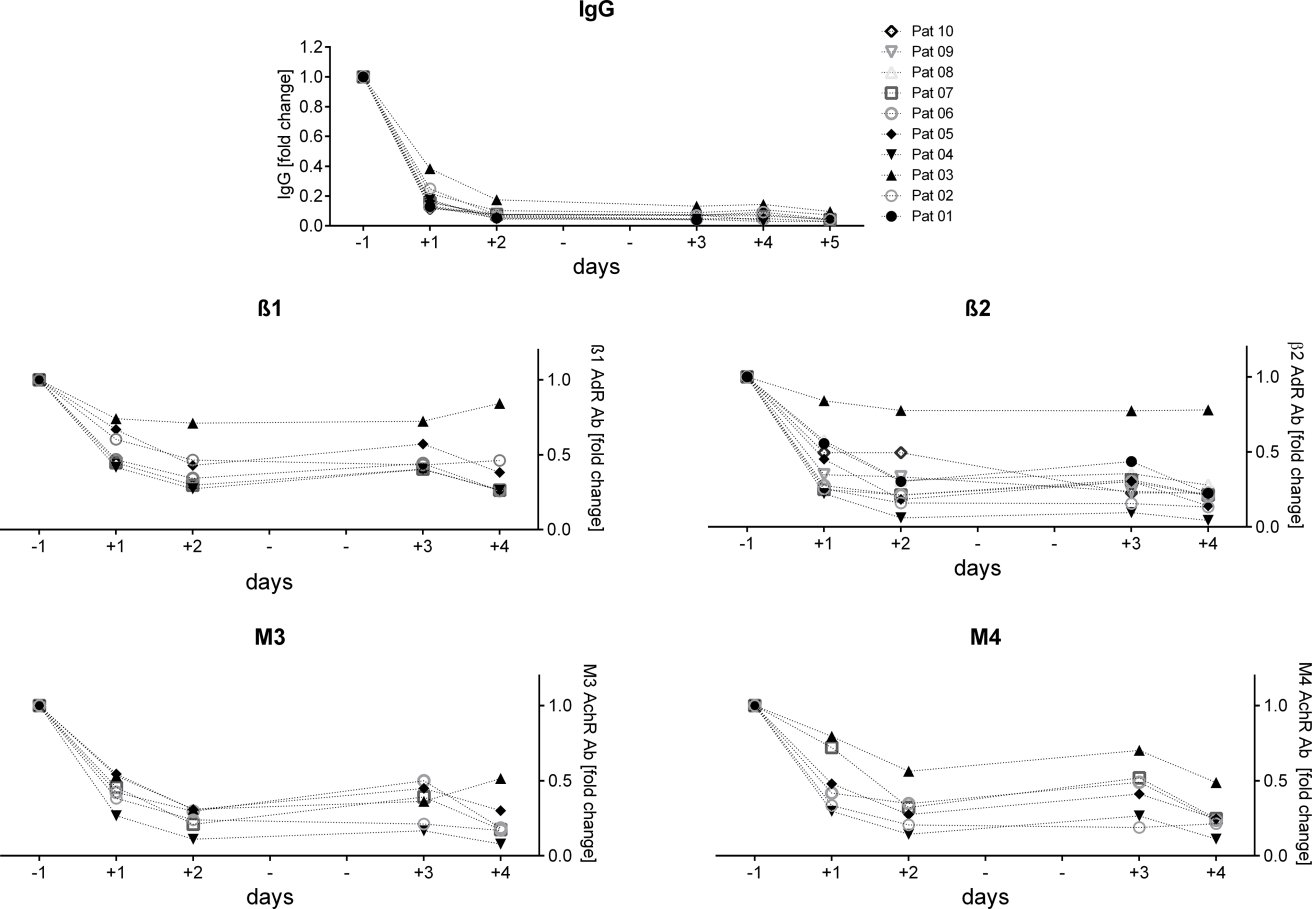
IgG and specific antibody levels during treatment. Total and specific ß1, ß2, M3 and M4 IgG in the serum before and during IA. The levels are depicted as x-fold change to day -1 level for each single patient days +1 (day after 1^st^ IA cycle) up to day +4 (day after the 4^th^ IA cycle).

In all patients total IgG levels were within the normal range (7–16 g/l) before and strongly reduced already after the first IA. After the 4^th^ IA, the IgG median was 0.73 g/l (range 0.21 to 1.74 g/l). IgA and IgM levels remained unchanged. The levels of total IgG and of ß2 IgG during IA is shown in all patients, and of ß1, M3 and M4 IgG in patients with pretreatment elevated levels in Fig 1. The ß1, ß2, M3 and M4 antibodies dropped parallel to the total IgG. Patient 3 was the only patient in whom the elevated ß1 and ß2 IgG showed only a minor decrease. To assess the efficacy of IA to remove ß2 we analyzed levels in the eluate from the 1^st^ IA. ß2 levels were mean 2-fold (ranging from 0.3–10-fold) higher in the eluate than in the serum at the day before IA in all patients and 1.1 fold in patient 3.

**Fig 2:**
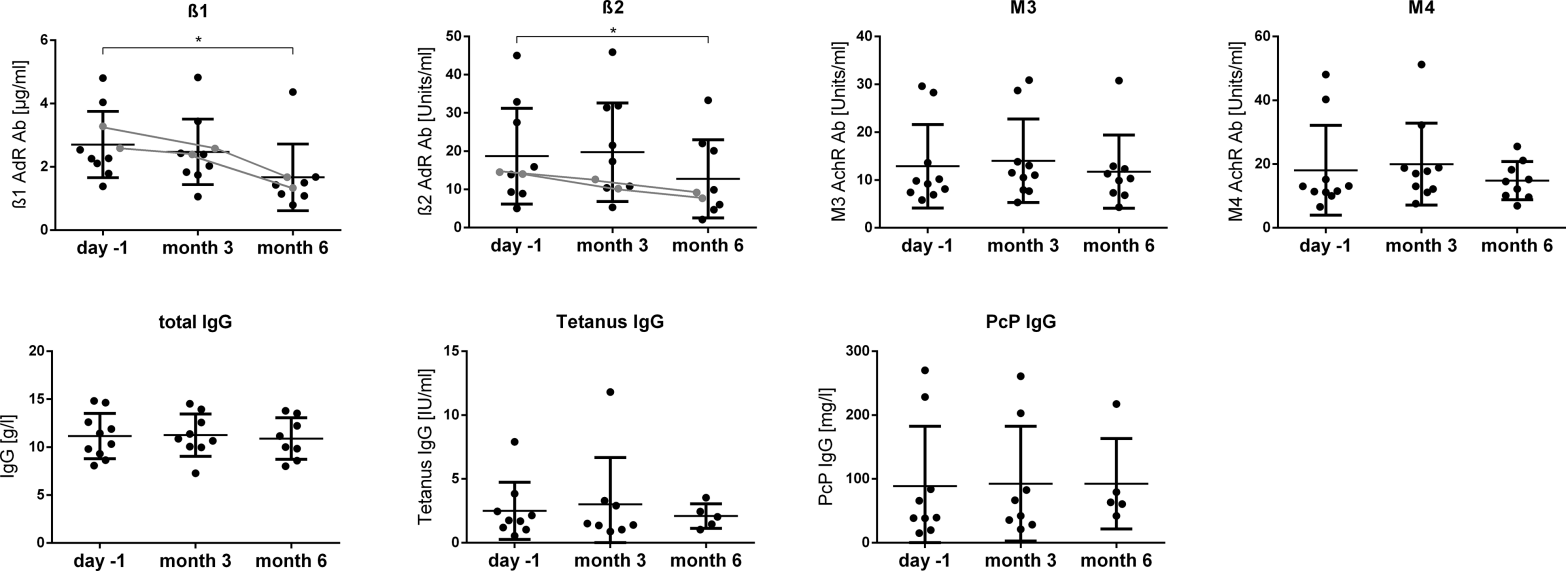
IgG and specific antibody levels follow-up. ß1, ß2, M3 and M4 IgG, total IgG, tetanus IgG, and pneumococcal IgG in the serum before and after 3 and 6 months of IA. The values are shown for each single patient. The course of ß1 and ß2 values of patients 4 and 5 with long term response are indicated by a line. In patient 9 ß2 antibodies were elevated at screening 3 months prior to study inclusion, but below the upper normal value (8.5 U) at the day before IA.

The levels of ß1, ß2 and M3 and M4 antibodies at months 3 and 6 are shown in Fig 2. Levels of ß1 and ß2 IgG were significantly lower at month 6 compared to pretreatment. In contrast, levels ​​of total IgG and the specific IgG against tetanus and pneumococcal polysaccharide after 3 and 6 months remained unchanged.

### Clinical course

**Fig 3:**
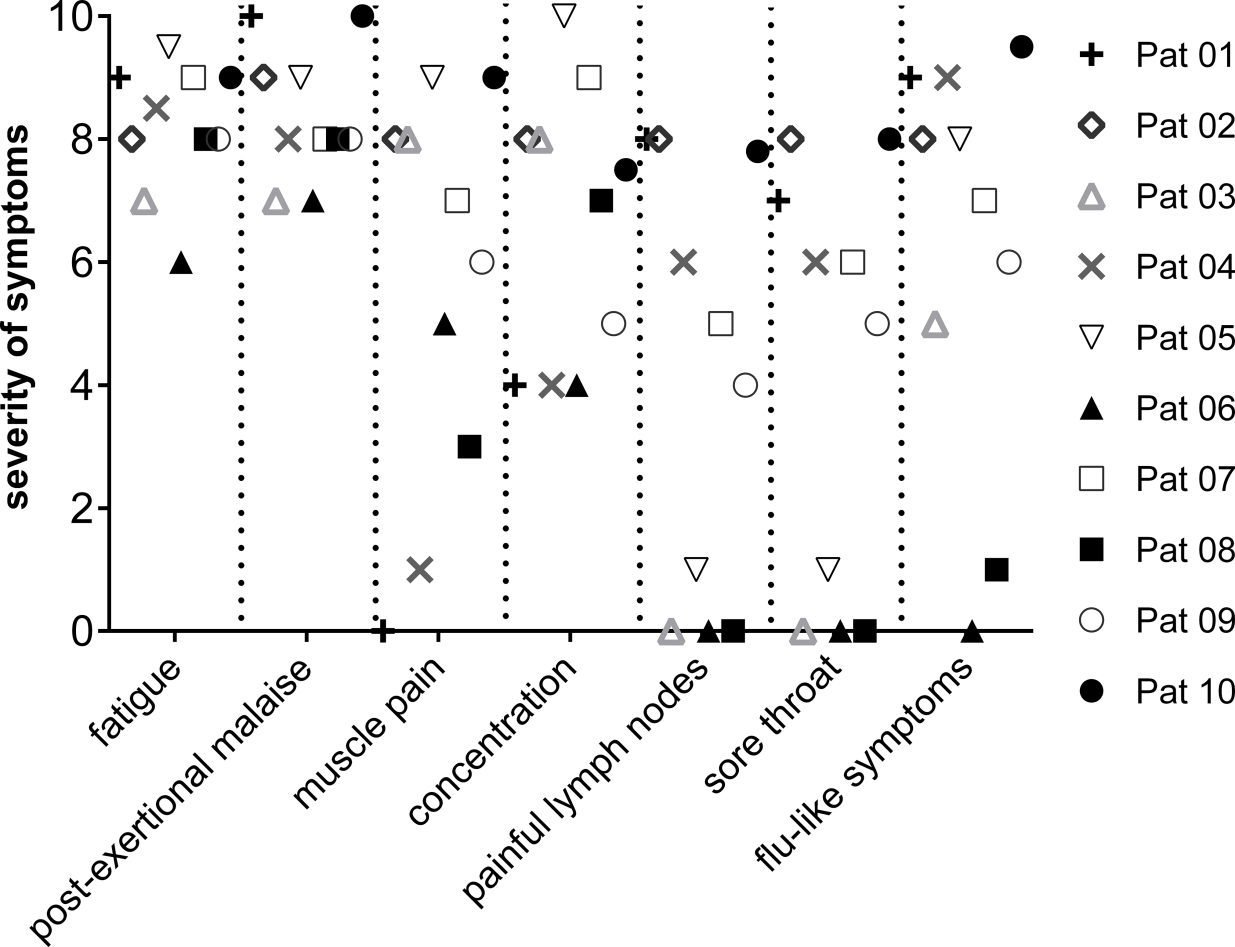
Patients condition before treatment. Symptom scores before IA. Symptoms are indicated as 0 (absent) to 10 (most severe).

At study onset all patients suffered from severe fatigue, post-exertional malaise and impaired concentration. Baseline symptom severity is shown in Fig 3. Further in a subset of patients moderate to severe (grade 3–10) muscle pain (n = 8), and the immune related symptoms painful lymph nodes (n = 6), sore throat (n = 6), and flu-like symptoms (n = 8) were present. Severity of functional impairment assessed by Bell score ranged from 10–50 (median 30, healthy 100) and of fatigue assessed by FACT-F ranged from 4–31 (mean 13, healthy 52) [16, 17].

**Fig 4:**
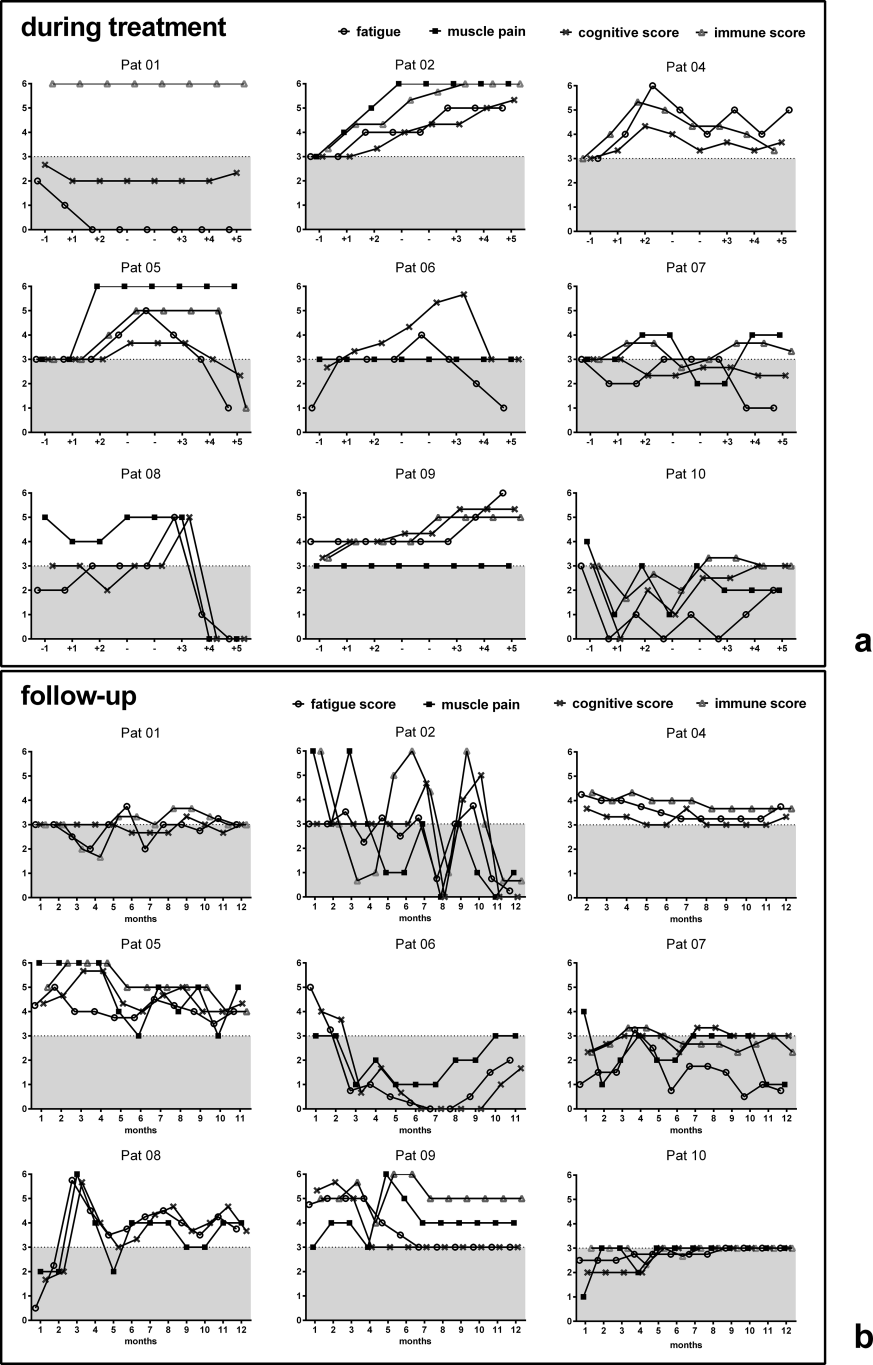
Development of symptoms. Symptom scores for fatigue, cognitive score, muscle pain and immune score during IA (A), and after 1 to 12 months of IA (B) are shown for each patient (3 unchanged, 4 slight, 5 marked improvement, 6 complete disappearance, 2 slight increase, 1 marked increase). The line indicates level 3 for unchanged symptoms.

The course of fatigue, concentration, muscle pain and immune symptoms during IA is shown for 9 patients in Fig 4A. A rapid improvement of several symptoms was reported by 7 of these 9 patients during IA. However, none of the patients completely recovered and 5 patients had worsening of fatigue towards the end of treatment despite improvement of other symptoms (patients 1, 5, 6, 7, and 8). Patient 10 reported worsening of all symptoms and patient 3 neither improvement nor worsening of symptoms (from patient 3 the data is not available).

**Fig 5:**
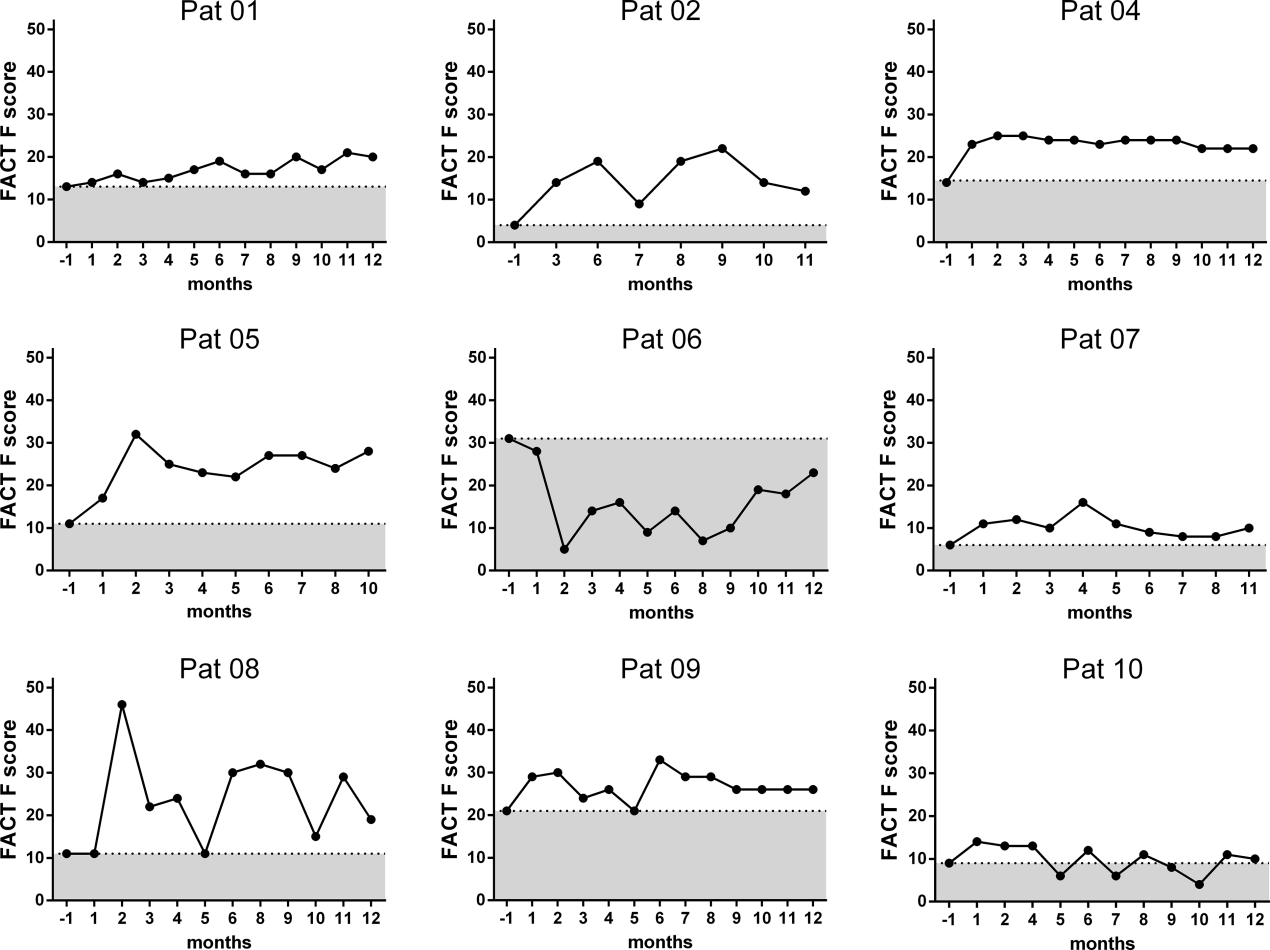
Fact score follow-up. FACT-F score assessing fatigue before and after 1 to 12 months of IA for each patient. The line indicates the individual pretreatment FACT-F score.

As follow up the CFS symptom score and FACT-F questionnaires were filled in by the patients monthly (Figs 4B and 5). Three patients reported improvement of fatigue and other symptoms for at least 12 months (patients 4, 5 and 9, Fig 4B). 2 patients had only short improvement lasting for one week (patient 1) and 6 weeks (patient 6) and 2 patients improved for several months following initial worsening (patient 2 and 8). Patient 8 had the most remarkable course. While she could hardly walk due to severe muscle fatigue before IA, she could walk several hundred meters at the last day of IA. After transient worsening of symptoms she then completely recovered for 7 weeks. Patient 6 had improvement of fatigue and cognitive symptoms for 6 weeks and went back to work, but then relapsed with severe post-exertional malaise and disease worsening for 6 months. Two patients had short improvement of symptoms during IA with complete disappearance of immune symptoms (pat. 1) and almost complete resolution of all symptoms (pat. 2). After IA both patients deteriorated following a respiratory tract infection and patient 2 had a fluctuating course thereafter. Patients 3, 7 and 10 did not improve during IA (from patient 3 the data is not available). FACT-F assessing severity of fatigue significantly improved from mean 13 before IA to mean 17.3 at month 3 and mean 20.7 at month 6 (p = 0.038 month 3 and p = 0.045 month 6 compared to before treatment).

### Functional assessment

**Fig 6:**
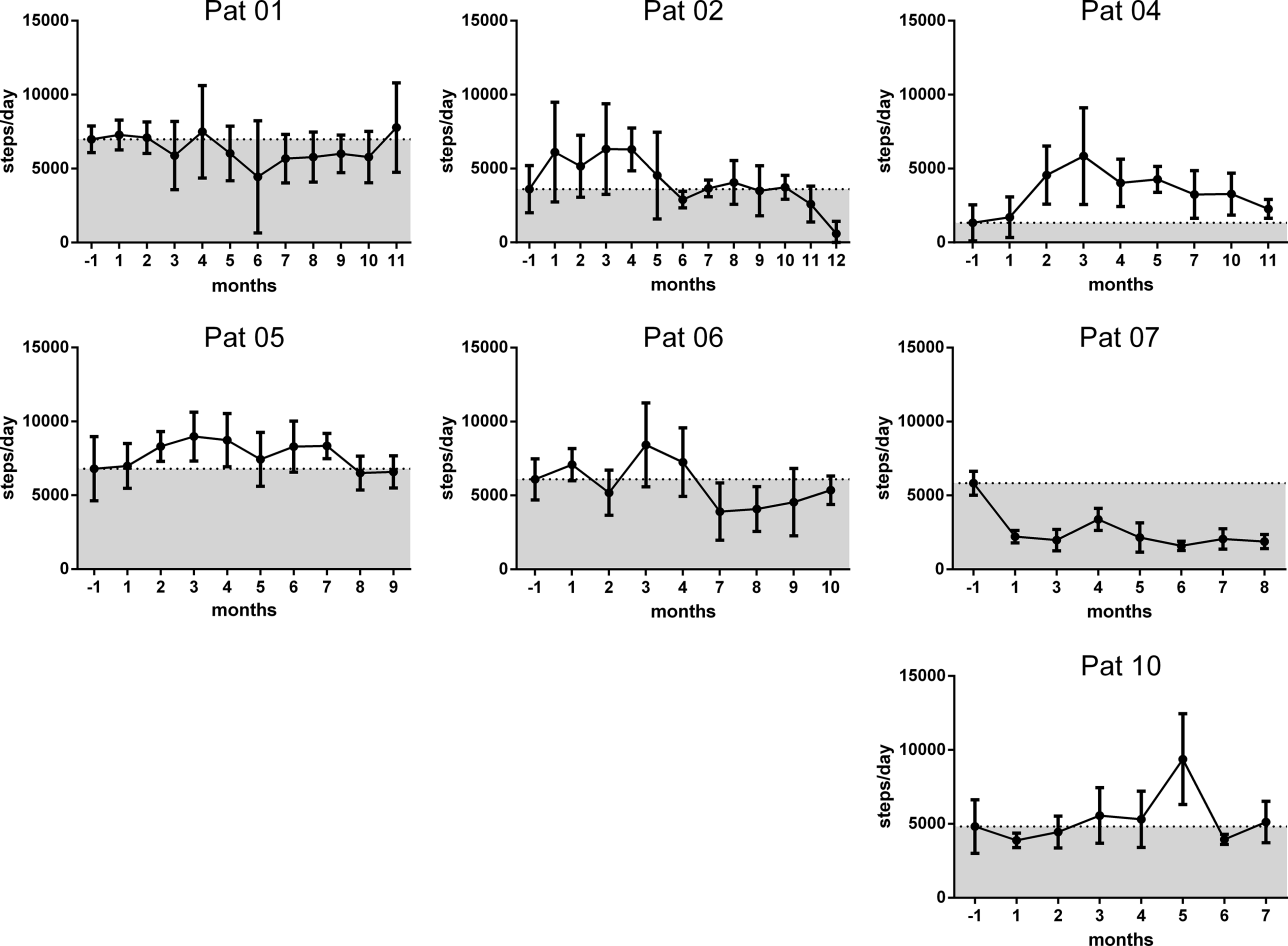
Patients mobility. Mean daily number of steps counted during one week before and thereafter monthly after IA for 7 patients (patients 1, 2, 4, 5, 6, 7 and 10).

In all patients we tried to objectively assess symptoms by measuring endothelial cell function and muscle strength. We could not observe a change neither in endothelial cell function nor pinch or hand grip at months 3 and 6 compared to pretreatment (data shown in S1 Fig supporting information material). Further, the numbers of steps were assessed for a week each month by a Vivofit activity tracker. Patients 4 and 5 showed improvement in numbers of steps after IA, while the steps in the other patients varied (Fig 6). Patients 8 and 9 could not be evaluated because the tracker was lost or did not record the pretreatment evaluation, respectively.

### B-cell phenotype

**Fig 7:**
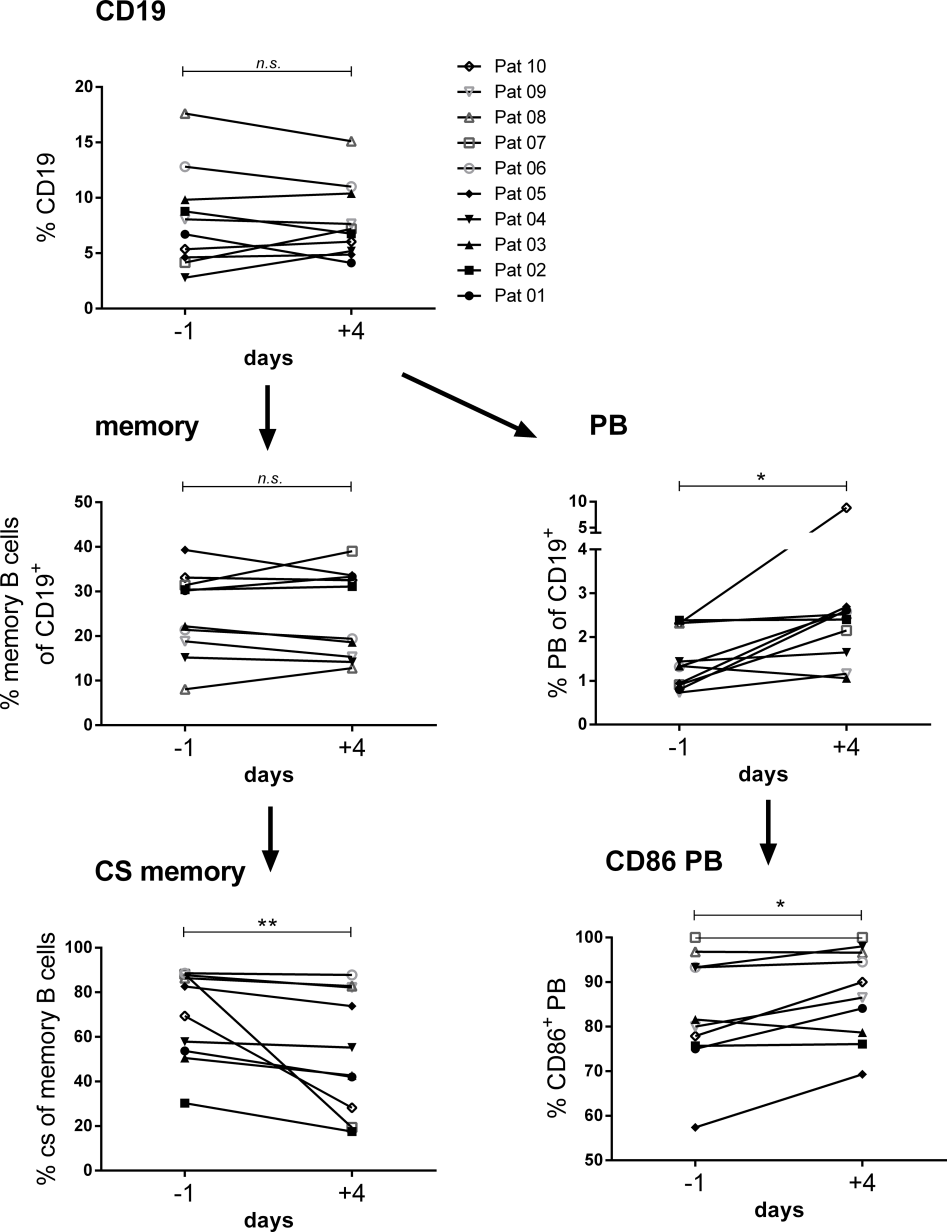
B cell subset analysis. Frequencies of CD19 total B cells, CD19+CD27+ memory B cells, CD19+CD27+IgM negative class switched (CS) B cells, plasmablasts (PB) and CD86+ plasmablasts before IA (day -1) and after the 4th IA (day -4).

We performed a comprehensive flow cytometric B-cell phenotyping in all patients before IA and after the 4th day of IA (Fig 7). While there was no change in total CD19+ B cells or total memory B cells (CD19+CD27+) we observed a significant decrease in the percentage of class-switched memory B cells (CD19+CD27+IgM negative) accompanied by an increase in the percentage of plasmablasts (CD38+ IgM negative). Further the fraction of CD86+ activated plasmablasts increased significantly.

## Discussion

We performed a first proof of concept study in 10 patients with infection-triggered CFS who had elevated ß2 antibodies. We could observe a rapid improvement of several symptoms in 7 of 10 patients, a long-term improvement in 3 patients and a sustained decrease of ß2 IgG levels. This observation is important in two respects. First it opens a perspective for a novel treatment with rapid onset of efficacy. Secondly, it provides further evidence that autoantibodies play a role in CFS in line with the results from the Norwegian rituximab trials [2–4].

IA is usually well tolerated in various types of disease [7–13, 15]. However, CFS patients are very sensitive to stress and environmental changes and frequently suffer from endothelial dysfunction and are thus more sensitive to alterations in plasma volume and electrolyte composition [20]. Further a hallmark of CFS is the exacerbation of symptoms due to exertion [1]. Therefore, we expected IA to be less well tolerated in CFS. Several patients indeed experienced aggravation of fatigue during IA. As described already for the Globaffin columns, we observed that IA is not fully selective as albumin and fibrinogen levels decreased as well requiring close monitoring.

Fatigue and other symptoms frequently occurring in CFS were assessed with a CFS specific questionnaire developed by Fluge *et al*. for the assessment of response to treatment with rituximab [3]. Symptom scores had been used in IA trials for other neuro-immunological diseases as well [12, 13], but the self-reported symptom changes are of course subjective. We tried to assess objectively symptom improvement by various measures. While the tracking of steps using a Vivofit activity tracker correlated with the symptom improvement reported, no correlation with muscle power and endothelial dysfunction could be observed [18–20].

We observed a rapid improvement of symptoms in 7 patients during the IA. In 4 patients the improvement was of short duration and symptoms reoccurred within 1–6 weeks. However, 2 of these patients then improved again for several months following initial transient worsening. 3 patients had a sustained improvement of fatigue and several symptoms for at least 12 months. Our observations are in line with clinical results of IA in other diseases. Rapid improvement of symptoms with both short and sustained responses is reported in myasthenia gravis and other neuro-immunological diseases as well [9, 11, 13].

Levels of ß2 adrenergic antibodies were low to undetectable in 9 of 10 patients after the IA cycles. In patient 3 we could not achieve reduction of ß2 by IA despite enrichment of ß2 in the eluate. Thus, it is likely that this patient has high affinity ß2 antibodies, which could not be well mobilized from cellular binding. A significant decrease of ß2 antibodies at months 6 was observed including patients who had no improvement at this time point. There was, however, some association between ß2 levels and clinical course. Patient 3 who had no improvement was the only in whom elevated ß1 and ß2 IgG decreased less than 30% during IA. Patients 4 and 5 with long term response were the only patients in whom ß2 IgG had already declined at month 3 (indicated as line in Fig 2). The sustained decrease in ß2 IgG is in accordance with our observation in the Norwegian rituximab trial in which, in patients responding to therapy, pretreatment elevated ß2 antibodies remained low 9 months after the last rituximab infusion [4]. Similarly, a long-term decrease of ß1 autoantibodies is observed in patients with dilatative cardiomyopathy after IA and is associated with improved clinical outcomes [10, 15].

Levels of total IgG and tetanus and pneumococcal IgG were similar to pretreatment levels at months 3 and 6. To better understand the potential mechanism of a selective disappearance of autoantibodies following IA we performed a comprehensive B cell phenotyping and observed decreased frequencies of memory B cells and increased frequencies of plasmablasts after 4 cycles of IA compared to pretreatment. This suggests that a differentiation of memory B cells into plasmablasts occurs possibly as a result of the marked IgG depletion. A potential explanation for a long-term decline of autoantibodies could be that due to IgG depletion B cell differentiation is strongly stimulated with autoreactive B cells more prone to go into apoptosis. This hypothesis of apoptosis of autoreactive B cells and consecutive loss of short living autoreactive plasma cells would be in line with the observed decrease of ß1 and ß2 autoantibodies 6 months after IA. There are few studies on potential immunomodulatory effects of IA. A study by Ramlow *et al*. showed a decrease of B cells and increase of activated T cells in IA responders compared to non-responders [21].

We have no evidence from our study, of course, that removal of the ß2 IgG itself resulted in disease improvement as other yet unknown autoantibodies may exist. ß2 antibodies are not specific for CFS/ME but were shown to be elevated in various other diseases including arrhythmia, chronic regional pain syndrome, postural tachycardia, and others [7]. We have indirect evidence, however, that ß2 IgG plays a pathogenic role in CFS/ME due to the association with immune activation [4]. In our previous study we could show that CFS/ME patients with elevated ß2 antibodies had higher IgG1-3 levels, more frequent T cell activation, and elevated ANA and TPO antibodies [4]. In B cells enhanced production of IgG1 upon ß2 adrenergic stimulation was shown in several studies [22]. Thus, the association of autoantibodies with enhanced IgG levels we observed suggests agonistic effects of ß2 adrenergic antibodies on B cell receptors. In T cells it was shown that ß2 agonists can reduce IFNγ and IL-2 production and inhibit lymphocyte proliferation [23]. In monocytes ß2-adrenergic stimulation has inhibitory effects on LPS-induced monocyte TNF production [24]. However, the effect of adrenergic stimulation on immune cells is complex and depends on the activation status of immune cells. Thus, in vitro functional assays are required to clarify the effect of the ß2 autoantibodies. This is currently studied in an ongoing project.

Taken together, this pilot study provides evidence that IA can effectively remove ß2 and M3/M4 autoantibodies in CFS/ME and can result in rapid moderate to marked symptom improvement. Larger clinical trials of IA are warranted to obtain more evidence for the efficacy of IA in CFS/ME.

## Supporting information

S1 Fig Patients muscular and endothelial function. Assessment of muscular and endothelial function before and after treatment (one week, three months and six months). No significant changes were detected. Isometric pinch strength of the stronger hand was analyzed using the pinch dynamometer (Saehan Corporation, Korea). The highest of three pinch measurements was used for analyses [18]. Peripheral endothelial function was evaluated by pulse arterial tonometry (PAT) device (EndoPAT-2000, Itamar, Israel). Assessments were performed under standardized conditions after at least 15 minutes of supine rest in a quiet, air-conditioned room [19].
(TIF)
