# Supplementary figures and images for: Immunoadsorption to remove ß2 adrenergic receptor antibodies in Chronic Fatigue Syndrome CFS/ME
Source: PLoS One. 2018 Mar 15;13(3):e0193672. doi: 10.1371/journal.pone.0193672 (PMC5854315; doi:10.1371/journal.pone.0193672)

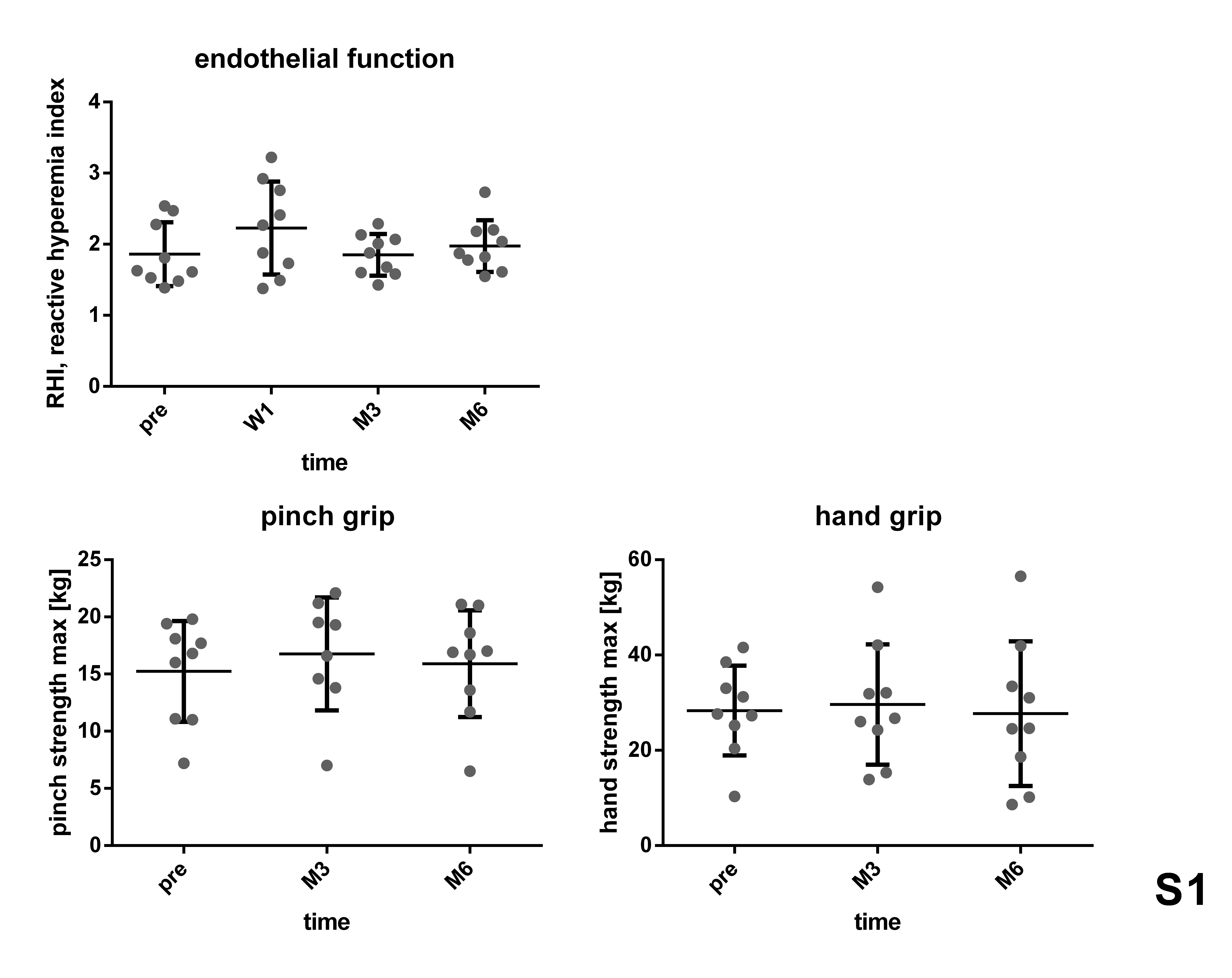

Supplement: S1 Fig — Assessment of muscular and endothelial function before and after treatment (one week, three months and six months). No significant changes were detected. Isometric pinch strength of the stronger hand was analyzed using the pinch dynamometer (Saehan Corporation, Korea). The highest of three pinch measurements was used for analyses [18]. Peripheral endothelial function was evaluated by pulse arterial tonometry (PAT) device (EndoPAT-2000, Itamar, Israel). Assessments were performed under standardized conditions after at least 15 minutes of supine rest in a quiet, air-conditioned room [19]. (TIF) [file pone.0193672.s001.tif]
